# Extensive Periprosthetic Lucencies and Acetabular Protrusio in a Hip Hemiarthroplasty: A Case Report

**DOI:** 10.7759/cureus.114488

**Published:** 2026-08-13

**Authors:** Jacob F Turner, Shabber Syed, Rameesha Shaheen, Nicholas Onyeador

**Affiliations:** 1 Radiology, Detroit Medical Center, Wayne State University, Detroit, USA; 2 Radiology, Michigan State University College of Osteopathic Medicine, Detroit, USA

**Keywords:** acetabular protrusio, hip hemi-arthroplasty, hip hemi-arthroplasty complications, particle disease, peri-prosthetic joint infection

## Abstract

Primary hip arthroplasties are common orthopedic procedures performed to replace a damaged or arthritic hip joint using prosthetic units. In the past several decades, the incidence of primary hip arthroplasty procedures has increased significantly. For many, the procedure provides long-term relief. However, a variety of complications can also arise, such as neurovascular injury, leg length discrepancy, infection, osteolysis, loosening, heterotopic ossification, and pseudobursa formation. Of these, several can result in failed hip arthroplasty, including aseptic loosening with osteolysis, infection, and instability. Imaging aids in differentiation between these causes of failed hip arthroplasty, which is crucial as there can be significant overlap in their clinical presentation.

This case report demonstrates a 74-year-old patient who had a remote history of left hip hemiarthroplasty that presented with severe periprosthetic lucencies and acetabular protrusio after a fall. The top of the differential diagnosis included aseptic loosening with periprosthetic osteolysis secondary to particle disease, periprosthetic joint infection, and traumatic fracture. Imaging played a vital role in achieving an accurate diagnosis and guiding management.

Modalities commonly utilized in the workup of arthroplasty complications include conventional radiography, computed tomography (CT), magnetic resonance imaging (MRI), and nuclear medicine techniques such as three-phase bone scans, white blood cell (WBC) scans, and fluorodeoxyglucose-positron emission tomography (FDG-PET). This article will discuss the importance of these modalities as part of a multifaceted approach to accurately diagnose arthroplasty complications.

## Introduction

Primary hip arthroplasties are common orthopedic procedures performed to replace a damaged or arthritic hip joint using prosthetic units. Hemiarthroplasty is one of the types of primary hip arthroplasty. These procedures can offer long-term pain relief and improved mobility, resulting in increased quality of life. In the United States, the incidence of primary hip arthroplasties increased 156% between the years 1996-2019 [1]. However, with the widespread increase of hip replacements performed around the world, there has also been a rise in postoperative complications and revision procedures. Possible postoperative complications include but are not limited to neurovascular injury, leg length discrepancy, infection, osteolysis, loosening, heterotopic ossification, and pseudobursa formation [2]. Of these, several can result in failed hip arthroplasty, including aseptic loosening with osteolysis, instability, and infection. Instability and infection tend to occur early in the postoperative course, whereas aseptic loosening with osteolysis tends to occur later [3]. Periprosthetic loosening can be septic or aseptic and, when suspected, warrants both a clinical examination and multimodal imaging workup [4].

Aseptic loosening with periprosthetic osteolysis can occur because of an entity known as particle disease. Particle disease is a term coined by Dr William Harris of Massachusetts General Hospital which describes an inflammatory host response to debris from the inserted prosthetics [3]. Small prosthetic particles provoke periprosthetic cells to express pro-inflammatory osteoclastic cytokines, which lead to increased activity of the osteoclasts and inhibited osteogenic activity of osteoblasts [3]. As will be demonstrated in the imaging of this case report, the increased activity of osteoclasts can result in osteolysis of periprosthetic bone. Patients may be asymptomatic or present with symptoms such as deep joint pain, instability, decreased range of motion, and/or joint swelling. It is important to note that while polyethylene particles are recognized as the major cause, particles from bone cement and metal can also result in this phenomenon [5]. The phenomenon of particle disease typically has an insidious clinical course.

Since periprosthetic joint infection (PJI) is one of the primary causes of a failed hip arthroplasty, it is important to keep on the differential. Implant-associated infection, especially when delayed-onset and low-grade, can be difficult to diagnose as patients may only present with loosening and persistent pain rather than clinical signs of infection [6]. Upon clinical presentation, clues that are considered pathognomonic for PJI include the presence of a draining sinus or an exposed implant [7]. However, these clues are absent in most cases. For these reasons, a multifaceted approach is often used for accurate diagnosis, including signs and symptoms, laboratory values, microbiology, and imaging.

In addition to the signs, symptoms, and imaging features of PJI, it is also important to be aware of the possible complications such as acetabular protrusio. Acetabular protrusio in the setting of PJI is a known entity found in the literature, and there should be a high index of suspicion for chronic or late PJI whenever acetabular protrusio occurs after hip arthroplasty [8]. However, particle disease and trauma may also result in acetabular protrusio.

There is currently a lack of consensus regarding periprosthetic joint infection timing. One of the available categorization systems, defined by Zimmerli et al., describes early periprosthetic infection as occurring less than three months post-surgery, delayed as between 3-24 months post-surgery, and late as greater than 24 months post-surgery [9]. The educational objectives of this case report of an uncommon case of culture-negative PJI with acetabular destruction and acetabular protrusio are to demonstrate and discuss key imaging features of PJI, some of which can appear similar to particle disease, and reinforce the utility of established diagnostic criteria for PJI.

## Case presentation

This case report examines a case of a 74-year-old female who presented after a fall with a remote surgical history of left hip hemiarthroplasty and left periprosthetic femur fracture requiring placement of cerclage cables and a structural allograft. Information regarding the year in which these procedures were performed and the type of prosthetic material used was unavailable, as the procedures were performed several years ago at an outside hospital. The patient reported severe (8/10) pain at the left hip with mild swelling, no weight-bearing ability, and was afebrile. The patient’s past medical history included hypertension, congestive heart failure, and obesity. Her lab values upon presentation are depicted in Table 1 and included a normal white blood cell (WBC) count of 6.5×10^³^/µL, an elevated erythrocyte sedimentation rate (ESR) of 40 mm/hr, and an elevated C-reactive protein (CRP) concentration of 2.14 mg/dL. These laboratory values were obtained on the same day that antibiotics were initiated and eight days before her eventual surgery.

**Table 1:** Laboratory values.

Lab Test	Patient Values	Reference Range
Absolute Eosinophil Count	0.1 x 10^3^/µL	0.0 to 0.6 x 10^3^/µL
Absolute Lymphocyte Count	0.6 x 10^3^/µL	1.0 - 3.8 x 10^3^/µL​​​​​​​
Absolute Monocyte Count	0.4 x 10^3^/µL​​​​​​​	0.1 - 0.88 x 10^3^/µL​​​​​​​
Absolute Neutrophil Count	5.2 x 10^3^/µL​​​​​​​	1.58 - 7.13 x 10^3^/µL​​​​​​​
Anion Gap	8 mMol/L	5 - 15 mMol/L
C-Reactive Protein (CRP)	2.14 mg/dL	0 - 0.5 mg/dL
Carbon Dioxide	25 mMol/L	21 - 31 mMol/L
Creatinine	0.49 mg/dL	0.6 - 1.2 mg/dL
eGFR (estimated Glomerular Filtration Rate)	98 mL/min/1.73 m²	60 - 120 mL/min/1.73 m²
Glucose	100 mg/dL	75 - 105 mg/dL
Hematocrit	34.90%	34.4% - 44.2%
Hemoglobin	11.5 gm/dL	11.5 - 15.1 gm/dL
Lactic Acid	1.6 mMol/L	0.4 - 2.0 mMol/L
Platelets	399 x 10^3^/µL​​​​​​​	150 - 450 x 10^3^/µL​​​​​​​
Potassium	3.3 mMol/L	3.5 - 5.1 mMol/L
RBC (Red Blood Cells)	4.08 x 10^6^/µL	3.91 - 5.15 x 10^6^​​​​​​​/µL
Red Cell Distribution Width (RDW)	19.10%	11.7% - 14.9%
ESR (Erythrocyte Sedimentation Rate)	40 mm/hr	0 - 20 mm/hr
Sodium	138 mMol/L	136 - 145 mMol/L
BUN (Blood Urea Nitrogen)	10 mg/dL	7 - 25 mg/dL
WBC (White Blood Cells)	6.5 x 10^3^	3.5 - 10.6 x 10^3^

**Figure 1:**
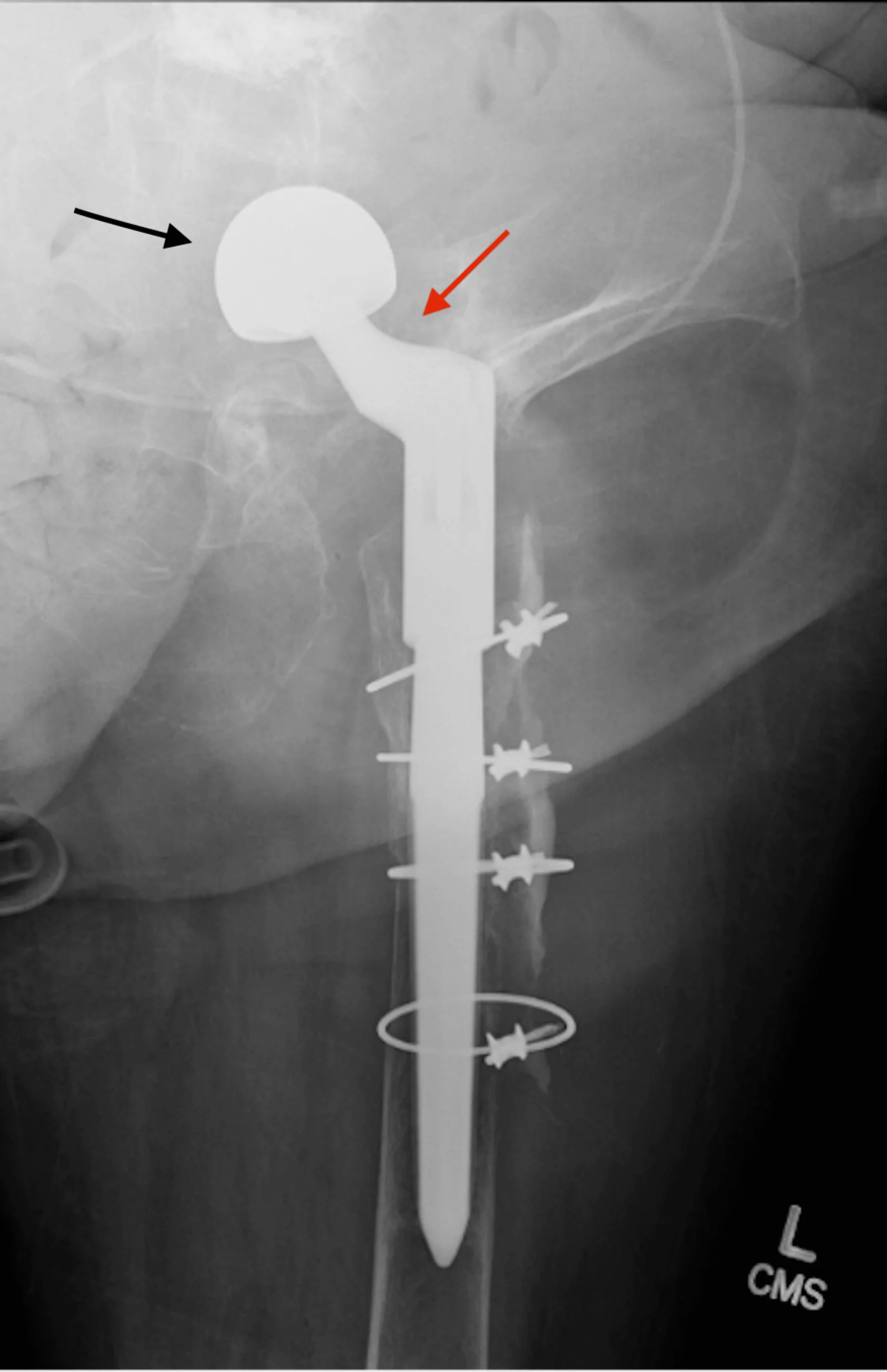
Anteroposterior (AP) radiograph of the left hip. The radiograph reveals periprosthetic lucencies with prosthetic femoral head (black arrow) and neck (red arrow) migrating superomedially through the acetabular wall.

The initial radiograph upon presentation revealed severe periprosthetic lucencies of the left hip hemiarthroplasty with erosion of the left acetabulum. The osteolysis involved the acetabulum, femoral neck, and proximal femoral diaphysis with severe cortical destruction predominantly at the acetabulum. The prosthetic femoral head and neck had migrated superomedially through the acetabular wall into the pelvis. There were no prior radiographs available to evaluate for progression (Figure 1).

**Figure 2:**
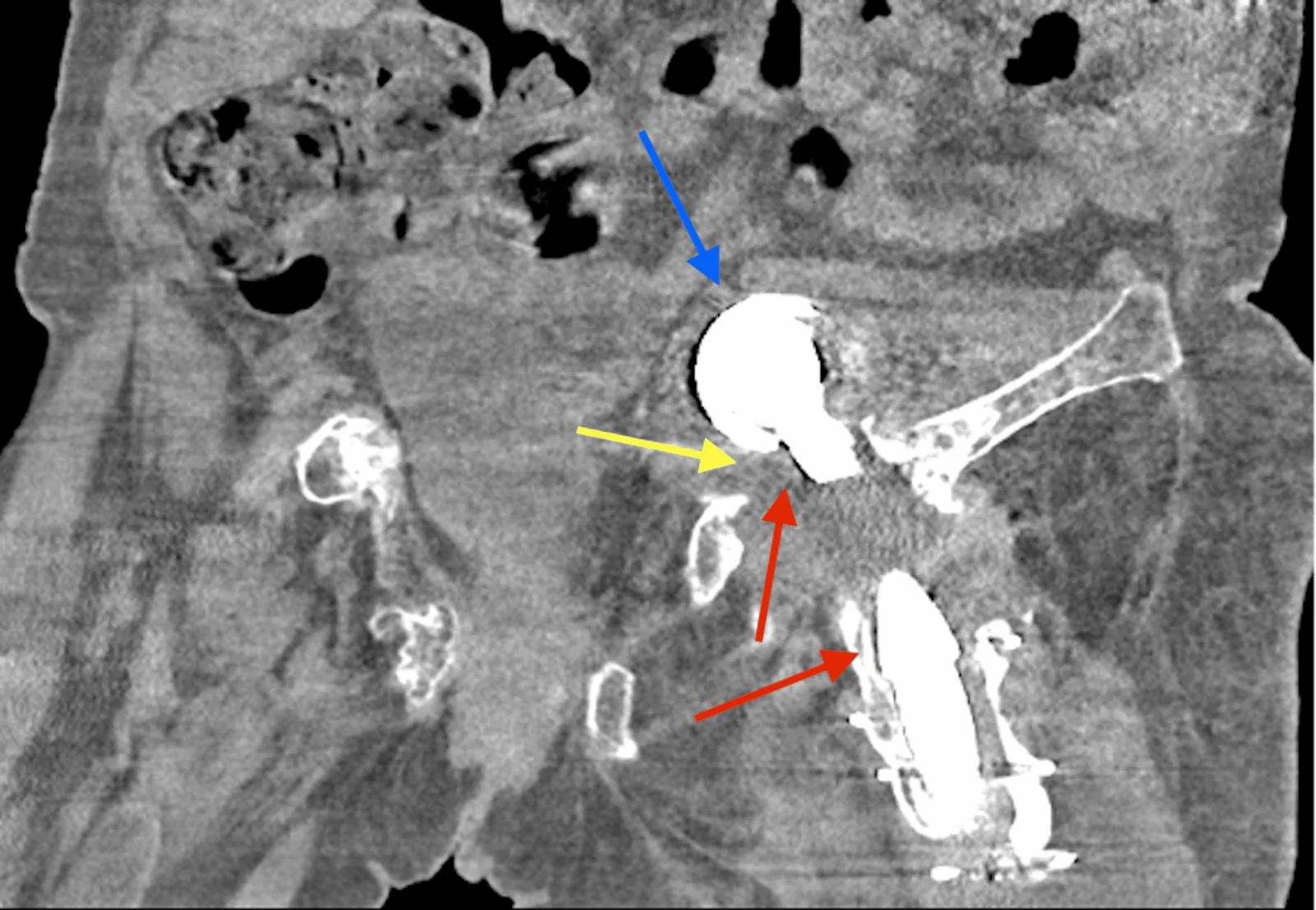
Coronal CT on soft tissue window. The image reveals an acetabular defect (yellow arrow), osteolysis (red arrows), and intrapelvic migration of the prosthetic femoral head and neck (blue arrow).

The initial CT also demonstrated severe periprosthetic lucencies of the left hip hemiarthroplasty, erosion of the left acetabulum, and acetabular protrusio, which are all represented in Figure 2. In addition, the initial CT showed two periprosthetic fluid collections; however, the evaluation was limited due to lack of intravenous contrast. At this time, the primary differential diagnosis included severe particle disease, periprosthetic joint infection, and traumatic fracture since there are overlapping features of these entities. All three could have resulted in prosthesis migration. Both PJI and traumatic fracture are known to typically cause an elevation in erythrocyte sedimentation rate (ESR) and C-reactive protein (CRP) levels. The severe acetabular erosion on imaging favored either PJI or particle disease as the etiology.

**Figure 3:**
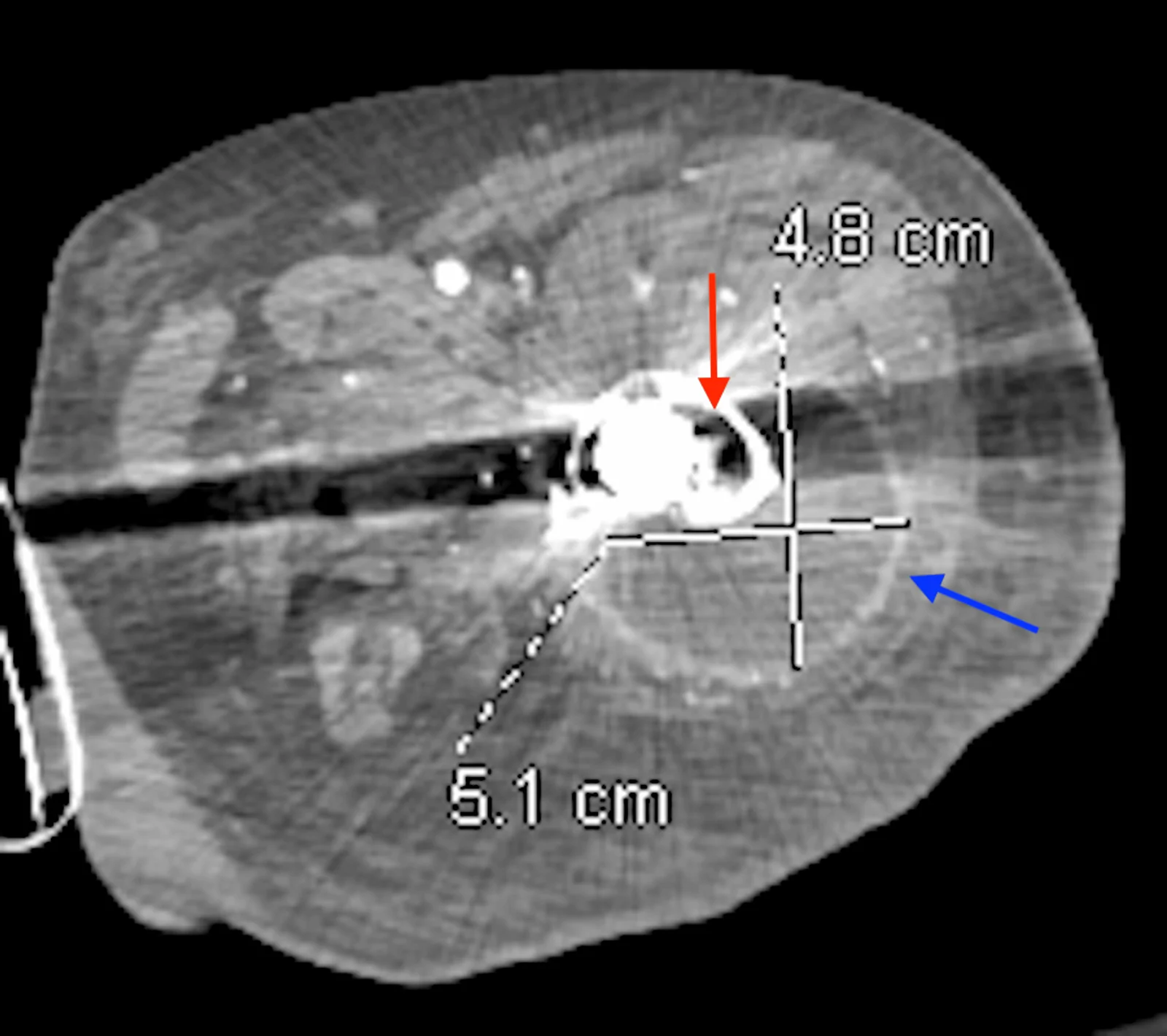
Axial CT angiography on soft tissue window. The image reveals left-sided periprosthetic lucencies (red arrow) and a fluid collection with an enhancing rim (blue arrow) directly posterolateral to the left proximal femoral diaphysis.

**Figure 4:**
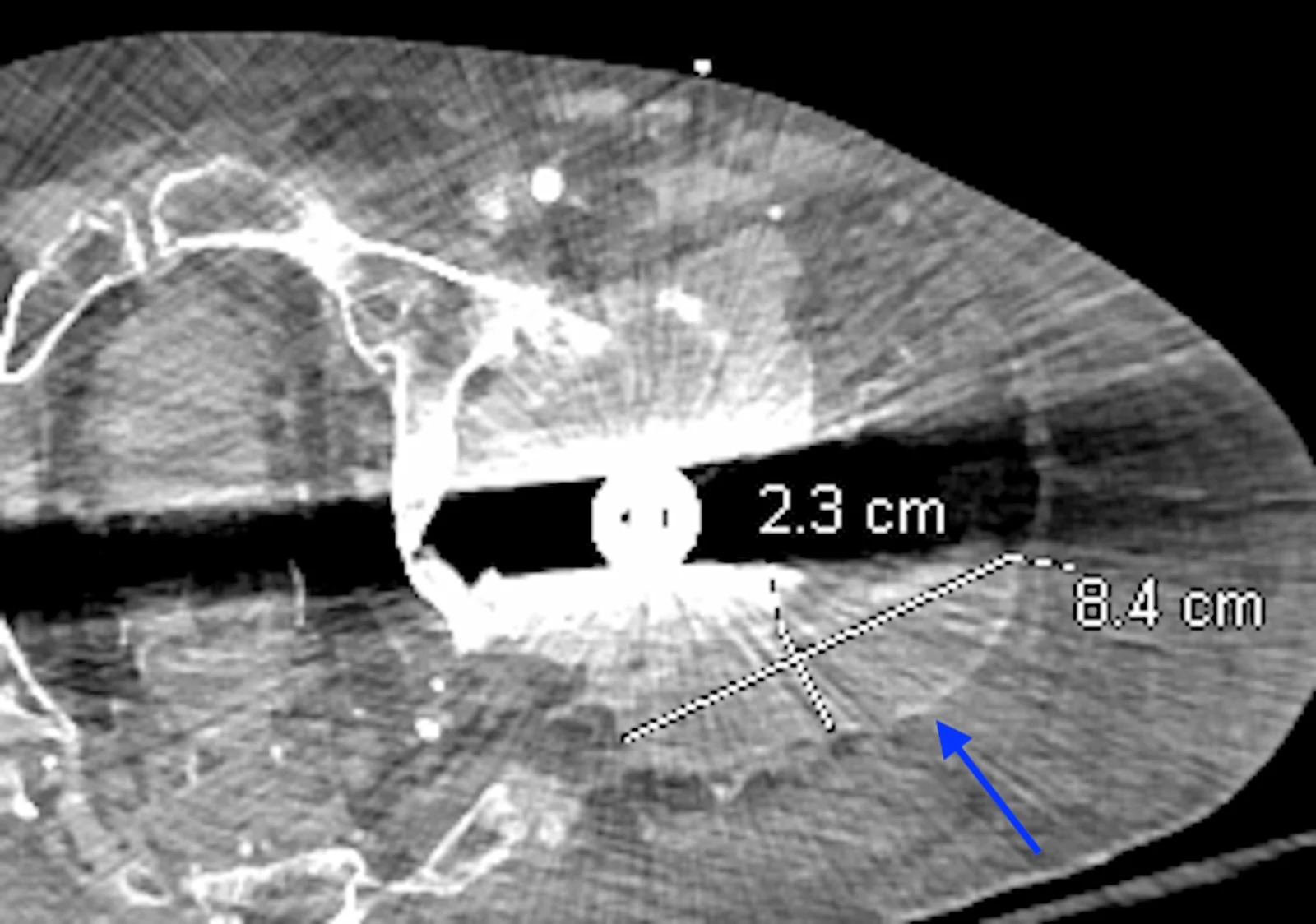
Axial CT angiography on soft tissue window. The image reveals an additional, separate fluid collection with an enhancing rim (blue arrow) posterior to the left acetabulum.

Subsequent CT angiography was performed to assess for vascular involvement from prosthetic migration and to further evaluate the fluid collections. The CT angiography showed two periprosthetic loculated fluid collections, concerning for abscesses, measuring 8.4 x 2.3 x 3.1 cm posterior to the acetabulum and 4.8 x 5.1 x 8.4 cm posterolateral to the left proximal femoral shaft. These periprosthetic fluid collections are depicted in Figure 3 and Figure 4. The patient was taken to the operating room, and approximately 100 mL of purulent fluid was found surrounding the hip joint, and the structural allograft femur bone had purulence around it. When applying the Parvizi criteria for periprosthetic joint infection, the patient fulfilled the pre-operative minor criteria of serum CRP > 1 mg/dL and ESR > 30 mm/hr. These serum CRP and ESR values, in addition to intra-operative findings of purulence, fulfilled diagnostic criteria for peri-prosthetic infection [10]. The patient required explantation of the prosthesis, removal of the infected structural allograft of the femur bone, and joint irrigation and debridement. 

Intra-operative wound cultures were obtained and remained negative after four days; however, the patient started receiving antibiotics eight days prior to the cultures being obtained. Two sets of two blood cultures were obtained; the first of which was obtained upon patient presentation and the other was obtained the day after the procedure. All of these blood cultures were negative after five days. In addition, intra-operative anaerobic cultures were obtained and remained negative after five days. Bone histopathology and implant sonication were not performed.

After the procedure, the infectious disease service was consulted. Despite negative intra-operative wound cultures, there was persistent clinical concern for osteomyelitis. As a result, intravenous vancomycin and ceftriaxone were then initiated as they are commonly selected agents for empiric treatment of osteomyelitis. This regimen was ordered to be given for a four-week duration, as four weeks is a reasonable length of treatment for surgically debrided osteomyelitis without retained hardware [11]. In addition, negative pressure wound therapy was utilized postoperatively. 

**Figure 5:**
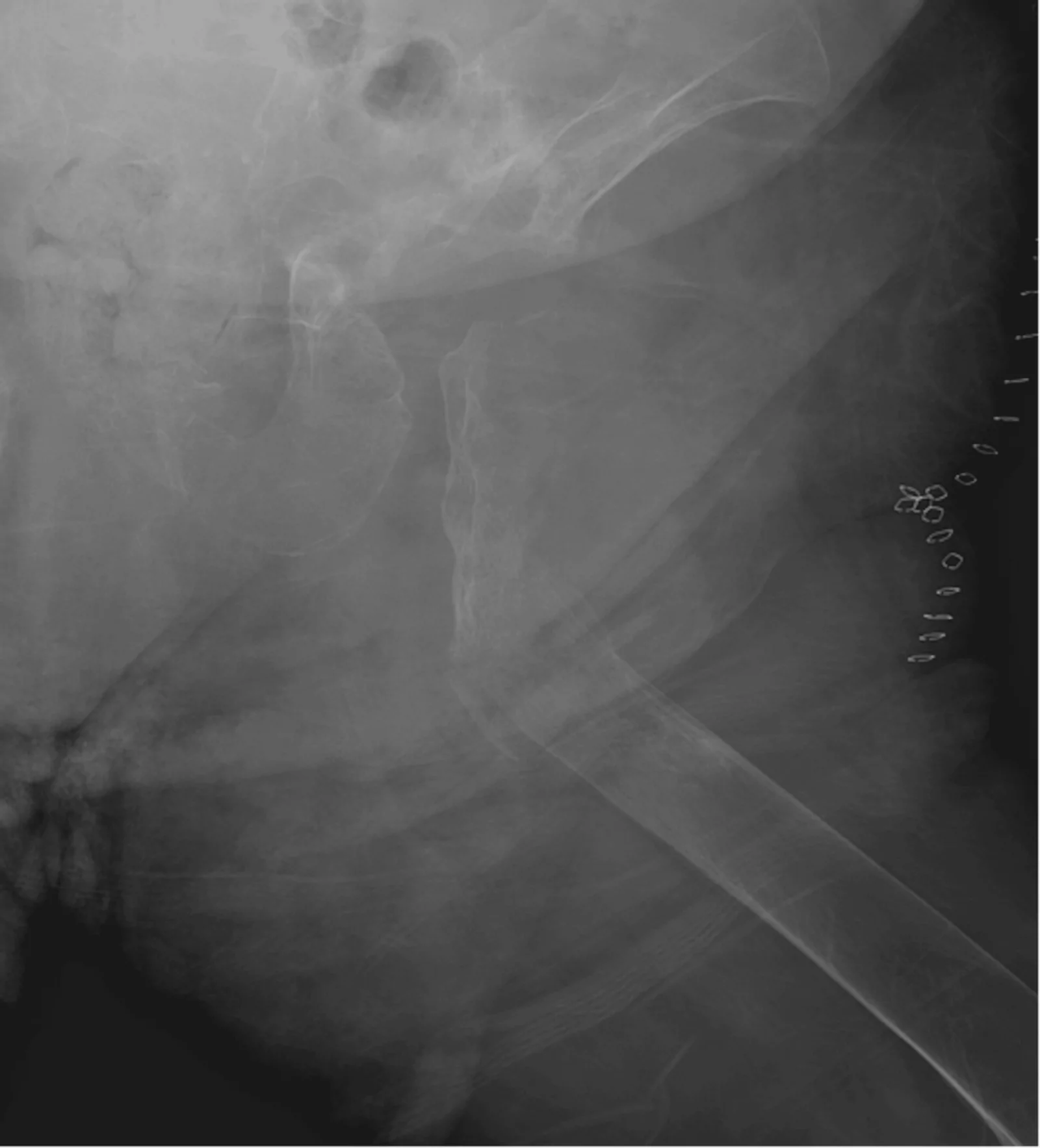
Anteroposterior (AP) radiograph of the left hip. The image reveals the removal of hip hemiarthroplasty hardware and osseous deformities of the left acetabulum and proximal femur.

A peripherally inserted central catheter was placed, and the patient was discharged to a skilled nursing facility. The patient was prescribed oxycodone for pain management at discharge. However, the following day the patient returned to the emergency department due to uncontrolled pain in her left hip. A left hip radiograph was obtained, which showed postoperative changes related to hip arthroplasty hardware removal with osseous deformities of the left acetabulum and femur as depicted in Figure 5. The patient’s pain regimen was escalated to consist of both oxycodone and extended-release morphine, and the patient was admitted for pain control. This escalation led to adequate pain control. After acceptance to a different skilled nursing facility, the patient was discharged and advised to follow up with the orthopedic surgeon who performed the explantation, joint irrigation, and debridement. There were no subsequent follow-up appointments or patient encounters; the patient was lost to follow-up.

## Discussion

There can be overlap in CT imaging features of aseptic loosening secondary to particle disease and periprosthetic infection. Both can present with focal periprosthetic lucencies, osteolysis, and joint effusion. CT is often ordered to aid in the detection of these features; however, differentiation between the two entities is not always possible with CT alone [6]. A key feature that can help differentiate particle disease from PJI on imaging is that particle disease will not produce a secondary bone response such as periosteal reaction [12]. The presence of rim-enhancing periprosthetic fluid collections in this case also supported the need for further evaluation for an infectious process.

As demonstrated in this case, PJI is important to diagnose as it can result in reduced function of the joint, reduced quality of life, and eventual arthrodesis, amputation, and resection of the implant. When there is clinical concern, obtaining labs such as white blood cell count, erythrocyte sedimentation rate, and C-reactive protein concentration is often the first step in workup. Typically, a patient with chronic periprosthetic infection will have an ESR > 30 mm/hr and/or a serum CRP > 1.0 mg/dL [13]. The patient in this case had an elevated ESR of 40 mm/hr and an elevated CRP of 2.14 mg/dL. In addition, it has been noted that many patients with a periprosthetic joint infection have a pre-operative serum WBC count within the normal range, similar to this case, and that pre-operative serum WBC count has a minimal role in diagnosing PJI [13]. Presence of various risk factors such as diabetes mellitus, corticosteroid use, obesity, chronic renal failure, immunocompromised state, and more can raise suspicion of periprosthetic joint infection [14]. 

CT, conventional radiography, single-photon emission CT (SPECT), bone scintigraphy, and MRI are all modalities used to aid in diagnosis of periprosthetic loosening. Periprosthetic lucencies >2 mm on CT help clue us in to possible loosening, making it important to evaluate for additional factors such as progression over time, morphology, and migration. A caveat to this is that in a symptomatic patient with periprosthetic lucency <2 mm, loosening cannot be excluded [4]. In our case, CT was the primary imaging modality used to aid in diagnosis. These CT’s revealed severe periprosthetic lucencies, erosion/osteolysis of the left acetabulum, and periprosthetic loculated fluid collections. The periprosthetic lucencies measured approximately 3 mm in thickness. The first CT was performed without intravenous contrast medium, which led to suboptimal evaluation of the soft tissue collections surrounding the prosthesis. The second CT was performed with intravenous contrast and demonstrated peripherally enhancing complex fluid collections, which were concerning for abscesses. The abscesses were then confirmed intra-operatively, supporting the leading diagnosis of periprosthetic infection as opposed to particle disease.

Conventional radiography may be used as a screening tool for hardware complications in patients with joint replacement; however they typically do not significantly aid in the differential diagnosis of PJI. In early periprosthetic infection, the majority of radiographs usually appear normal [15]. Nevertheless, radiographs are typically the first imaging study obtained as they may help in evaluation of degree of loosening, osteolysis, and detection of fractures [7]. The ability to distinguish periprosthetic infection from aseptic loosening is limited. Findings on radiographs that may suggest PJI include aggressive periostitis and gas formation, while a finding favoring particle disease includes multiple punctate metallic particles around the prosthesis [16]. However, these delineating findings are only found in a fraction of cases. Nonspecific signs on radiographs include implant loosening, periosteal reaction, and bone resorption. 

Although not utilized in our case, MRI and various nuclear medicine scans can be performed as part of the workup for periprosthetic infection. Three-phase bone scintigraphy, WBC scans, and FDG-PET scans are all considered highly sensitive; therefore, if negative, they can aid in exclusion of periprosthetic infection. Increased uptake on all phases of a three-phase bone scan (blood flow, blood pool, and delayed) is demonstrated in both periprosthetic infection and aseptic loosening; therefore is a nonspecific finding. A limitation of bone scintigraphy for evaluation of PJI is that false-positive results can occur up to approximately two years postoperatively due to normal bone remodelling [17]. However, three-phase bone scintigraphy has high negative predictive value, and a negative study helps to disfavor periprosthetic infection as the diagnosis. Ultimately, three-phase bone scintigraphy is viewed as a supplemental study in the workup of periprosthetic infection. 

Regarding leukocyte scintigraphy and FDG-PET for evaluation of periprosthetic hip infection, the techniques have been found to be comparable in sensitivity and specificity. Leukocyte scintigraphy has a sensitivity of 88% and specificity of 92%; FDG-PET has a sensitivity of 86% and specificity of 93%. When using these nuclear medicine techniques, it has been reported that a combined three-phase bone scan with leukocyte scintigraphy provides the highest specificity at 96% [18]. 

MRI can also be utilized to differentiate between PJI and aseptic loosening. Metal artefact reduction (MAR) sequences can be implemented to reduce the susceptibility artefact that commonly occurs from prostheses. On MRI, periprosthetic infection commonly demonstrates a lamellated, hyperintense synovium on fluid-intense sequences while aseptic loosening typically demonstrates a frond-like, hypertrophied synovium [19]. These classic synovial patterns, if present, may aid in differentiation of the two entities. Additional findings on MRI that may aid in evaluation are the periosteal reaction, lymphadenopathy, capsular edema, fluid collections, and fistulas that can be present in periprosthetic infection and are not typically seen in aseptic loosening [20]. While not utilized in this case, MRI may have added additional diagnostic value.

In the presented case, the patient was lost to follow-up after being discharged to her second skilled nursing facility. The absence of long-term clinical and radiographic follow-up is an important limitation when evaluating patient outcomes after explantation, debridement, and an antibiotic therapy regimen.

## Conclusions

Postoperative complications after hip hemiarthroplasty include periprosthetic infection and particle disease, which can have similar appearances on imaging. These entities can result in a failed arthroplasty and revision procedures, which makes it critical to correctly diagnose them. CT plays a major role in evaluating abnormalities in bones, organs, and soft tissues, but alone can be insufficient in reliably differentiating between aseptic loosening and periprosthetic infection. In this case, the initial CT without intravenous contrast showed osteolysis with periprosthetic lucencies and two fluid collections, which prompted a second CT with intravenous contrast for further evaluation. Correlating imaging findings with patient clinical presentation, lab studies, microbiology, and operative findings of purulence helped to provide a definitive diagnosis of culture-negative periprosthetic joint infection. Delayed diagnosis of this entity can result in significant patient morbidity. This uncommon case of culture-negative PJI with severe acetabular destruction and acetabular protrusio demonstrates important imaging features of PJI to recognize, some of which can appear similar to particle disease, and reinforces the utility of established diagnostic criteria for accurate diagnosis of PJI.
